# Modeling optimal treatment strategies in a heterogeneous mixing model

**DOI:** 10.1186/s12976-015-0026-x

**Published:** 2015-11-25

**Authors:** Seoyun Choe, Sunmi Lee

**Affiliations:** Department of Mathematics, Graduate School, Kyung Hee University, Seoul, 02447 Korea; Department of Applied Mathematics, Kyung Hee University, Yongin-si, 446-701 Korea

**Keywords:** A two-group influenza model, Heterogeneous mixing, Preferred mixing, Optimal control theory, Targeted treatment strategies

## Abstract

**Background:**

Many mathematical models assume random or homogeneous mixing for various infectious diseases. Homogeneous mixing can be generalized to mathematical models with multi-patches or age structure by incorporating contact matrices to capture the dynamics of the heterogeneously mixing populations. Contact or mixing patterns are difficult to measure in many infectious diseases including influenza. Mixing patterns are considered to be one of the critical factors for infectious disease modeling.

**Methods:**

A two-group influenza model is considered to evaluate the impact of heterogeneous mixing on the influenza transmission dynamics. Heterogeneous mixing between two groups with two different activity levels includes proportionate mixing, preferred mixing and like-with-like mixing. Furthermore, the optimal control problem is formulated in this two-group influenza model to identify the group-specific optimal treatment strategies at a minimal cost. We investigate group-specific optimal treatment strategies under various mixing scenarios.

**Results:**

The characteristics of the two-group influenza dynamics have been investigated in terms of the basic reproduction number and the final epidemic size under various mixing scenarios. As the mixing patterns become proportionate mixing, the basic reproduction number becomes smaller; however, the final epidemic size becomes larger. This is due to the fact that the number of infected people increases only slightly in the higher activity level group, while the number of infected people increases more significantly in the lower activity level group. Our results indicate that more intensive treatment of both groups at the early stage is the most effective treatment regardless of the mixing scenario. However, proportionate mixing requires more treated cases for all combinations of different group activity levels and group population sizes.

**Conclusions:**

Mixing patterns can play a critical role in the effectiveness of optimal treatments. As the mixing becomes more like-with-like mixing, treating the higher activity group in the population is almost as effective as treating the entire populations since it reduces the number of disease cases effectively but only requires similar treatments. The gain becomes more pronounced as the basic reproduction number increases. This can be a critical issue which must be considered for future pandemic influenza interventions, especially when there are limited resources available.

## Background

The timely and effective countermeasures of influenza challenge global health experts around the world, especially when limited resources are available. Mathematical modeling has made significant contributions to understanding the spread of influenza, and also providing useful insights to control or decrease the disease burden [1–4]. A number of mathematical models assume random or homogeneous mixing for the influenza dynamics, which can provide a good approximation to real epidemiological phenomenon [5, 6]. This simple assumption of homogeneous mixing can be extended to more general mathematical models with multi-patches or age structure by incorporating contact matrices to capture the dynamics of the heterogeneously mixing populations [1, 7]. In general, contact or mixing patterns are difficult to measure in many infectious diseases including influenza. There is no doubt that mixing patterns are considered to be one of the critical factors for infectious disease modeling.

There are many different approaches that allow us to investigate the impact of contact patterns on the transmission dynamics of infectious diseases. The age-dependent contact matrices based on empirical social data have been estimated [8, 9]. Age-dependent transmission matrices that describe the mixing and the probability of infection are studied using synthetic data [10]. In these studies, it has been noted that contact patterns are strongly dependent on distinct age groups, and therefore, the heterogeneity of contact patterns should be recognized as an important feature for the realistic modeling of many infectious diseases. Moreover, individual based models or network based models can provide more details on the disease dynamics by studying the effects of heterogeneous and clustered contact patterns. Contact patterns and their underlying network structures have shown to be one of the critical factors for determining the characteristics of infectious disease transmission [11, 12]. Also, several different heterogeneity types in infectious disease models have been incorporated, such as susceptibility, infectivity and mixing patterns [13, 14]. Agent or network based models have been developed to study effective controls in the influenza pandemic [2, 15, 16].

Deterministic models which are much simpler than network based models have been successfully employed to study the transmission dynamics of various infectious diseases and continued to produce valuable insights. Preferred mixing has been used to highlight the role of contact patterns in the HIV transmission dynamics [17, 18]. The impact of selective mixing is studied in the transmission of STDs [19]. In these studies, the contact rate matrices are formulated in terms of activity levels and subpopulation sizes by using a proportionate mixing assumption. The relation between the basic reproduction number and the initial exponential growth rate of an epidemic to models with heterogeneous mixing has been studied [20–22]. The authors show that an epidemic with heterogeneous mixing may have a quite different epidemic size than an epidemic with homogeneous mixing, even though they may have the same reproduction number and initial exponential growth rate. Determination of the final size of an epidemic under the assumption of heterogeneous mixing requires additional data from the initial exponential growth stage of the epidemic [21]. More recently, a two-group influenza model has been used to study the impact of heterogeneous mixing on the probability of the extinction of influenza [23, 24]. It has been pointed out that heterogeneous mixing between two subgroups would play a key role to explain the delays in the geographic spread of the 2009 H1N1 pandemic observed in Mexico and Japan.

In this manuscript, a deterministic two-group model is used to study the influenza transmission dynamics in heterogeneous environments. A two-group model allows for different activity levels and heterogeneous mixing between subgroups. In particular, two groups are coupled by a mixing matrix whose entries *p*_*ij*_, *i, j*=1,2 represent the proportion of individuals in group *i* that contact individuals in the other group *j*. This two-group model of influenza is an extension of the prototype model by Brauer [20] in which a two-group model is used to investigate the impact of proportionate mixing on the basic reproduction number and the final epidemic size. Now, our model involves more extensive heterogeneous mixing scenarios between the two groups. Specifically, several mixing scenarios are considered, including proportionate mixing, preferred mixing and like-with-like mixing, by varying the group mixing fractions.

We explore how this mixing pattern can affect the basic reproduction number and the final epidemic size. Heterogeneous mixing certainly changes the reproduction number and the final epidemic size, but it is not trivial to determine whether different mixing assumptions can change them substantially or not. The level of transmissibility measured by the basic reproduction number $\mathcal {R}_{0}$ is varied to highlight the differences and similarities for the results under several heterogeneous mixing scenarios. Moreover, we formulate an optimal control framework to investigate how these mixing patterns will influence the effectiveness of group-specific treatment strategies in the two-group model. Under various mixing scenarios, optimal group-specific treatment strategies and the corresponding influenza outcomes are compared. This can help us address some of the important issues such as allocating optimal treatments for future pandemic preparedness plans.

## Methods

### A heterogeneous mixing model

We consider a two-group influenza model based on a standard compartmental SITR model. Two additional compartments, a latent class and an asymptomatic class, are included due to the characteristics of influenza. Each class is divided into two subpopulations of sizes *N*_1_ and *N*_2_. For each group *i*=1,2, we have a susceptible class *S*_*i*_, a latent class *L*_*i*_, an infected class with symptoms *I*_*i*_, an asymptomatic infected class without symptoms *A*_*i*_ and a treated class *T*_*i*_. A two-group influenza model involves two different age groups, which are connected by a mixing matrix (*p*_*ij*_) for *i,j*=1,2, by allowing for the possibility of subgroups with different activity levels and heterogeneous mixing between these subgroups. A two-group influenza model with two subgroups can be written as

(1) $$ \begin{aligned} S_{1}'&=-a_{1}\left[p_{11}\frac{S_{1}(I_{1}+\sigma T_{1}+\delta A_{1})}{N_{1}}+p_{12}\frac{S_{1}(I_{2}+\sigma T_{2}+\delta A_{2})}{N_{2}}\right],\\ L_{1}'&=a_{1}\left[p_{11}\frac{S_{1}(I_{1}+\sigma T_{1}+\delta A_{1})}{N_{1}}+p_{12}\frac{S_{1}(I_{2}+\sigma T_{2}+\delta A_{2})}{N_{2}}\right]-\kappa_{1}L_{1},\\ I_{1}'&=p\kappa_{1}L_{1}-(\alpha_{1}+u_{1})I_{1},\\ A_{1}'&=(1-p)\kappa_{1}L_{1}-\eta_{1}A_{1},\\ T_{1}'&=u_{1}I_{1}-\alpha_{T,1}T_{1},\\ D_{1}'&=d_{1}\alpha_{1}I_{1}+d_{T,1}\alpha_{T,1}T_{1},\\ \end{aligned}  $$

$$ \begin{aligned} S_{2}'&=-a_{2}\left[p_{21}\frac{S_{2}(I_{1}+\sigma T_{1}+\delta A_{1})}{N_{1}}+p_{22}\frac{S_{2}(I_{2}+\sigma T_{2}+\delta A_{2})}{N_{2}}\right],\\ L_{2}'&=a_{2}\left[p_{21}\frac{S_{2}(I_{1}+\sigma T_{1}+\delta A_{1})}{N_{1}}+p_{22}\frac{S_{2}(I_{2}+\sigma T_{2}+\delta A_{2})}{N_{2}}\right] -\kappa_{2}L_{2},\\ I_{2}'&=p\kappa_{2}L_{2}-(\alpha_{2}+u_{2})I_{2},\\ A_{2}'&=(1-p)\kappa_{2}L_{2}-\eta_{2}A_{2},\\ T_{2}'&=u_{2}I_{2}-\alpha_{T,2}T_{2},\\ D_{2}'&=d_{2}\alpha_{2}I_{2}+d_{T,2}\alpha_{T,2}T_{2}. \end{aligned}  $$

For each group *i*, *κ*_*i*_ is the rate of passage from the latent to the symptomatic infective or asymptomatic infective classes; *p* is the fraction of latent members who become symptomatic infectious, and the fraction (1−*p*) progress to the asymptomatic stage; *δ* is the infectivity reduction factor for the asymptomatic class and *σ* is the infectivity reduction factor for treated members; *α*_*i*_(*α*_*T,i*_) is the natural recovery rate from the infected (treated) to the removed stage and *η*_*i*_ is the rate of passage from the asymptomatic to the removed stage. Also, *u*_*i*_ is a constant treatment rate, which will be modified as a time-dependent treatment rate in the next section. *d*_*i*_ (*d*_*T,i*_) is the disease-induced death rate from the infected class (the treated class).

In this manuscript, we generalize the proportional mixing assumption to the preferred mixing one and we carry out mathematical analysis under different mixing patterns. Suppose that the members of group *i* make *a*_*i*_ contacts per unit time and that the fraction of contacts made by the members of group *i* with the members of group *j* is *p*_*ij*_, for *i, j*=1,2; then we have the following:

$$ p_{11} + p_{12} = p_{21} + p_{22} = 1. $$

For our two-group influenza model, we consider preferred mixing, in which a fraction *π*_*i*_ of each group mixes randomly with its own group and the remaining members mix proportionately. Thus, preferred mixing is given by

(2) $$ \begin{aligned} p_{11} &= \pi_{1} + (1-\pi_{1})p_{1},&&&&p_{12} = (1-\pi_{1})p_{2},\\ p_{21} &= (1-\pi_{2})p_{1}, &&&&p_{22} = \pi_{2}+(1-\pi_{2})p_{2},\\ \end{aligned}  $$

where

$$ \begin{aligned} p_{i} = \frac{(1-\pi_{i})a_{i} N_{i}} {(1-\pi_{1})a_{1} N_{1} + (1-\pi_{2})a_{2} N_{2}}. \end{aligned} $$

More details on the preferred mixing formulation can be found in previous studies [18, 20].

### The impact of mixing patterns on the contact matrix

Let us investigate the impact of different mixing patterns on the contact matrix. The contact matrix is defined as the product of group activity level, *a*_*i*_ and the group mixing proportions *p*_*ij*_ (*i,j*=1,2) given in (2).

$$ C = \left[ \begin{array}{cc} a_{1}p_{11} & a_{1}p_{12} \\ a_{2}p_{21} & a_{2}p_{22} \end{array} \right]. $$

To illustrate the impact of different mixing patterns, several group mixing fractions are chosen; *C*_1_ (*π*_1_=*π*_2_=0), *C*_2_ (*π*_1_=0.25,*π*_2_=0.75), *C*_3_ (*π*_1_=*π*_2_=0.5), *C*_4_ (*π*_1_=0.75,*π*_2_=0.25) and *C*_5_ (*π*_1_=*π*_2_=1) using *a*_1_=0.5260,*a*_2_=0.2670 and *N*_1_=1750 and *N*_2_=250. Then, we get the following contact matrices:

$$ C_{1} = \left[ \begin{array}{cc} 0.4904 & 0.0356 \\ 0.2489 & 0.0181 \end{array} \right], C_{2} = \left[ \begin{array}{cc} 0.5167 & 0.0093 \\ 0.0652 & 0.2018 \end{array} \right], C_{3} = \left[ \begin{array}{cc} 0.5082 & 0.0178 \\ 0.1245 & 0.1425 \end{array} \right], $$

$$ C_{4} = \left[ \begin{array}{cc} 0.5025 & 0.0235 \\ 0.1645 & 0.1025 \end{array} \right], C_{5} = \left[ \begin{array}{cc} 0.5260 & 0 \\ 0 & 0.2670 \end{array} \right]. $$

When the group mixing fraction is *π*_1_=*π*_2_=0, we have proportionate mixing which is a special case of preferred mixing (*C*_1_). It is also possible to have like-with-like mixing when *π*_1_=*π*_2_=1, in which members of each group mixes only with members of the same group. That is, for like-with-like mixing, *p*_11_=*p*_22_=1 and *p*_12_=*p*_21_=0 (*C*_5_). For like-with-like mixing, the contact matrix is a diagonal matrix.

### The basic reproduction number

One of the most important factors in mathematical epidemiology is the basic reproduction number, which is the average number of secondary infectious cases when one infectious individual is introduced to a whole susceptible population. The basic reproduction number can be calculated by using the next generation matrix approach, outlined in [25, 26]. Since the model includes treatments, we also compute the controlled reproduction number in the presence of constant treatment rates (*u*_*i*_).

Let **x**=(*L*_1_,*I*_1_,*A*_1_,*T*_1_,*L*_2_,*I*_2_,*A*_2_,*T*_2_)^*T*^ and *F*(**x**) represent all the new infection rates. The net transition rates out of the corresponding compartment are represented by *V*(**x**). Then, we find the Jacobian matrix of **F**(**x**) and **V**(**x**) evaluated at the disease-free equilibrium point **x**^∗^, which consists of *S*_1_=*N*_1_,*S*_2_=*N*_2_ and the rest of the components zero. The spectral radius of the matrix **FV**^−1^ yields the basic reproduction number ($\mathcal {R}_{0}$) and the controlled reproduction number ($\mathcal {R}_{c}$) in the presence of treatments (more details are given in Appendix A).

As a result, the basic reproduction number $\mathcal {R}_{0}$ with *u*_1_=*u*_2_=0 is

$$ \mathcal{R}_{0}=\frac{1}{2}\left(a_{1}p_{11}\Phi_{1}+a_{2}p_{22}\Phi_{2}+\sqrt{(a_{1}p_{11} \Phi_{1}-a_{2}p_{22}\Phi_{2})^{2}+4a_{1}a_{2}p_{12}p_{21}\Phi_{1}\Phi_{2}} \right), $$

where $\Phi _{1}=\left (\frac {\delta (1-p)}{\eta _{1}}+\frac {p}{\alpha _{1}} \right), \Phi _{2}=\left (\frac {\delta (1-p)}{\eta _{2}}+\frac {p}{\alpha _{2}} \right)$.

The controlled reproduction number $\mathcal {R}_{c}$ is

$$ \mathcal{R}_{c}=\frac{1}{2}\left(a_{1}p_{11}\Gamma_{1}+a_{2}p_{22}\Gamma_{2}+\sqrt{(a_{1}p_{11}\Gamma_{1}-a_{2}p_{22}\Gamma_{2})^{2}+4a_{1}a_{2}p_{12}p_{21}\Gamma_{1}\Gamma_{2}} \right), $$

where $\Gamma _{1}=\left (\frac {\delta (1-p)}{\eta _{1}}+\frac {p(\alpha _{T,1}+\sigma u_{1})}{\alpha _{T,1}(\alpha _{1}+u_{1})} \right), \Gamma _{2}=\left (\frac {\delta (1-p)}{\eta _{2}} + \frac {p(\alpha _{T,2}+\sigma u_{2})}{\alpha _{T,2}(\alpha _{2}+u_{2})} \right)$.

The expressions for the basic reproduction number $\mathcal {R}_{0}$ and the controlled reproduction number $\mathcal {R}_{c}$ have been generalized from the ones with proportional mixing [20] to the ones with preferred mixing. The activity levels and the mixing fractions play a critical role in the basic reproduction number.

For instance, taking partial derivatives of *p*_*ij*_ with respect to *π*_*i*_, we can show that *p*_11_ and *p*_22_ increase and *p*_12_ and *p*_21_ decrease as either *π*_1_ or *π*_2_ increase to 1. This results in the basic reproduction number increasing as preferred mixing becomes like-with-like mixing. Numerical sensitivity analysis of ${\mathcal {R}}_{0}$ and ${\mathcal {R}}_{c}$ is carried out in the next section.

### The final size relation

For a one-group epidemic model, there is a final size relation that makes it possible to calculate the size of the epidemic from the reproduction number [5, 22, 27, 28]. In this section, we establish a final size relation for the two-group model (1) with *u*_1_=*u*_2_=0. This relation does not involve the basic reproduction number explicitly but still makes it possible to calculate the size of the epidemic from the model parameters. The final size relation of the model (1) can be obtained as

(3) $$ \left[ \begin{array}{c} \ln\frac{S_{1}(0)}{S_{1}(\infty)} \\ \ln\frac{S_{2}(0)}{S_{2}(\infty)} \end{array} \right] = \left[ \begin{array}{c} a_{1} p_{11}\Phi_{1}\left(1-\frac{S_{1}(\infty)}{N_{1}(0)} \right)+ a_{1} p_{12}\Phi_{2}\left(1-\frac{S_{2}(\infty)}{N_{2}(0)}\right) \\ a_{2} p_{21}\Phi_{1}\left(1-\frac{S_{1}(\infty)}{N_{1}(0)} \right)+ a_{2} p_{22}\Phi_{2}\left(1-\frac{S_{2}(\infty)}{N_{2}(0)}\right) \end{array} \right].  $$

For the relation between the final size relation and the basic reproduction number, we use the eigenvector **v** of $\mathcal {R}_{0}$ as in [21], then

(4) $$ \mathbf{v}=[v_{1},0,0,0,v_{2},0,0,0]^{T},  $$

where

$$\begin{array}{@{}rcl@{}} v_{1}=\frac{a_{1} p_{11} \Phi_{1} -a_{2} p_{22} \Phi_{2} \sqrt{(a_{1}p_{11}\Phi_{1} -a_{2}p_{22}\Phi_{2})^{2}+4a_{1}a_{2}p_{12}p_{21}\Phi_{1}\Phi_{2}}}{2a_{1} p_{2} \Phi_{1} (1-\pi_{2})}, v_{2}=1. \end{array} $$

The eigenvalue and the eigenvector can be written as

(5) $$ \left[ \begin{array}{cc} p_{11} \Phi_{1} v_{1} & (1-\pi_{1})p_{1} \Phi_{2} v_{2} \\ (1-\pi_{2})p_{2} \Phi_{1} v_{1} & p_{22} \Phi_{2} v_{2} \end{array} \right]\left[ \begin{array}{c} a_{1} \\ a_{2} \end{array}\right] = \left[\begin{array}{c} \mathcal{R}_{0} v_{1} \\ \mathcal{R}_{0} v_{2} \end{array} \right].  $$

Also, the activity levels can be found in terms of $\mathcal {R}_{0}$ using (4) and (5),

$${}a_{1}=\mathcal{R}_{0}\left(\!\frac{v_{1}p_{22}-v_{2}(1-\pi_{1})p_{1}}{p_{11}p_{22}-p_{12}p_{21}}\!\right)\left(\!\frac{1}{\Phi_{1} v_{1}}\!\right),a_{2}=\mathcal{R}_{0}\left(\frac{v_{2}p_{11}-v_{1}(1-\pi_{2})p_{2}}{p_{11}p_{22}-p_{12}p_{21}}\right)\left(\frac{1}{\Phi_{2} v_{2}}\right). $$

When these values are substituted into the final sized system, *S*_1_(*∞*) and *S*_2_(*∞*) can be expressed in terms of the model parameters. As seen in the analytic expression above, the group specific final sizes are coupled with each other in a complex way. In the previous study [21], it can be simplified under proportionate mixing and shown that the final epidemic size in group 1 is larger than in group 2 when *a*_1_>*a*_2_. Also, it has been pointed out that $\mathcal {R}_{0}$ alone is not enough to determine the final epidemic size due to this complex coupling. It is difficult to observe how different mixing patterns affect the final size relation. Therefore, we carry out sensitivity analysis numerically as mixing patterns are varied in the following section. The details on the computation of the final size relation are given in Appendix A and the references [20, 21].

## Modeling optimal treatment strategy

Optimal control theory has been used frequently in a number of biological and epidemiological models (see [29] and the references therein). For influenza transmission models, optimal interventions are identified and the impact of optimal interventions on the influenza dynamics are investigated [30–32]. Various intervention strategies such as vaccination, antiviral treatment, and isolation controls are studied; optimal strategies for the 1918 influenza pandemic with limited resources [33] and age-dependent optimal vaccination strategies are investigated in context of the transmission dynamics of the 2009 influenza pandemic [34, 35].

We employ optimal control theory to explore the impact of antiviral treatment in situations that mimic 1918-like influenza pandemic scenarios. We modify model (1) by incorporating time-dependent control functions to measure the effectiveness of group-specific treatment strategies. Intervention strategies (policies) are modeled by the functions *u*_*i*_(*t*)(*i*=1,2) that externally control the number of treated cases. The objective functional $\mathcal {F}$ over a finite time interval [ 0,*T*] is given by the expression:

(6) $$\begin{array}{@{}rcl@{}} \mathcal{F}(u_{1}(t),u_{2}(t)) = {\int_{0}^{T}} \left(C_{1}I_{1}(t)+C_{2}I_{2}(t)+\frac{W_{1}}{2}{u_{1}^{2}}(t)+\frac{W_{2}}{2}{u_{2}^{2}}(t)\right) {dt}. \end{array} $$

We choose to model the control efforts via a linear combination of quadratic terms, *u*_*i*_^2^(*t*)(*i*=1,2). The constants *C*_1_,*C*_2_ are the weight constants for infected individuals and *W*_1_,*W*_2_ are the relative costs of the interventions. We might include the cost of deaths in the objective functional so that we would emphasize the cost of disease-induced deaths. However, it turns out that the results including the cost of deaths and the ones without the cost of deaths are almost indistinguishable (results are not shown here).

The optimal control problem is that of finding optimal functions (*u*_1_^∗^(*t*),*u*_2_^∗^(*t*)) such that

(7) $$\begin{array}{@{}rcl@{}} \mathcal {F}(u_{1}^{*}(t), u_{2}^{*}(t)) = min_{\Omega} \mathcal {F}(u_{1}(t),u_{2}(t)), \end{array} $$

where *Ω*={(*u*_1_(*t*),*u*_2_(*t*))∈(*L*^1^(0,*T*))^2^∥0≤*u*_1_(*t*),*u*_2_(*t*)≤*b, t*∈ [ 0,*T*]} subject to the state equations given by (1) with initial conditions. The existence of optimal controls is guaranteed from standard results on optimal control theory [36]. Pontryagin’s Maximum Principle is used to establish necessary conditions that must be satisfied by an optimal solution [37]. Derivations of the necessary conditions are shown in Appendix B.

A two point boundary method [29] is employed to find numerical solutions to (7). First, the state system (1) is solved forward with initial conditions. Then, the adjoint system with transversality conditions is solved backward in time. Finally, the optimality condition is updated and whole steps are iterated until convergence is achieved. The baseline parameter values are given in Table 1, which has been taken [20].

**Table 1:** Parameter definitions and baseline values used in the numerical simulations

Parameter	Description	Values
*a*_*i*_	Group-specific activity level for age group *i*	0.1−0.84
*π*_*i*_	Group-specific mixing fraction for age group *i*	0−1
*α*_*i*_	Recovery rate for infected class for age group *i* (days ^−1^)	0.244
*η*_*i*_	Rate of progression from asymptomatic to recovered class for age group *i* (days ^−1^)	0.244
*α*_*T,i*_	Recovery rate for treated class for age group *i* (days ^−1^)	0.323
*κ*_*i*_	Rate of progression from latent to infective or asymptomatic class (days ^−1^)	0.526
*p*	Fraction of latent individuals who become infected	0.667
*σ*	Infectivity reduction for treated class	0.2
*δ*	Infectivity reduction for asymptomatic class	0.5
*d*_*i*_	Mortality rate for infected class for age group *i* (days ^−1^)	0.01 (*i*=1)
		0.08 (*i*=2)
*d*_*T,i*_	Mortality rate for recovered class for age group *i* (days ^−1^)	0.005 (*i*=1)
		0.04 (*i*=2)
*b*	The upper bound of control	0.2, 0.5, 1
*C*_*i*_	Weight constants on *I*_*i*_(*i*=1,2)	1
*W*_*i*_	Weight constants on controls (*i*=1,2)	1,100
*N*_1_	Population size for group 1	1750
*N*_2_	Population size for group 2	250

## Results and discussion

We present the numerical simulations associated with implementing optimal treatment control functions as well as their effect on two-group influenza dynamics under different mixing patterns. In order to investigate the impact of mixing patterns, the group mixing fractions *π*_*i*_ are varied from 0 to 1 for each *i*=1,2, including proportionate mixing (*π*_*i*_=0), half mixing (*π*_*i*_=0.5) and like-with-like mixing (*π*_*i*_=1).

### The results in the absence of treatments

**Fig. 1:**
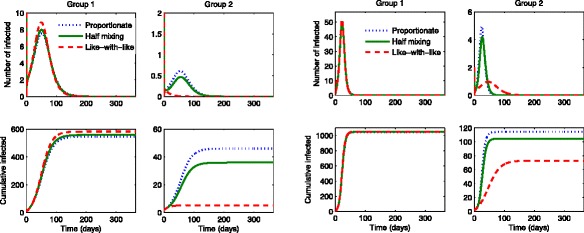
The impact of mixing patterns on the group-specific incidence. The number of incidence for each group is displayed under proportionate mixing (dotted), half mixing (solid) and like-with-like mixing (dashed). The *left panels* show the results for the moderate value of $\mathcal {R}_{0}=1.32$, while the *right panels* show the results for the higher value of $\mathcal {R}_{0}=2.45$

First, we illustrate the influenza dynamics of (1) in the absence of treatments (*u*_1_=*u*_2_=0). Figure 1 compares the group specific incidence under three mixing scenarios: proportionate mixing (dotted curve), half mixing (solid curve) and like-with-like mixing (dashed curve). Furthermore, the results are shown under two different values of $\mathcal {R}_{0}$ (using a moderate value and a higher value on the left and right, respectively). The group 2 incidence is smaller under like-with-like mixing than the one using proportionate mixing, while the group 1 incidence is larger. The incidence in the lower activity level group 2 gets significantly larger as the mixing becomes more proportionate than the one in the higher activity group. Hence, this leads to the total incidence or the final epidemic size getting smaller as the mixing becomes like-with-like mixing. Also, it clearly shows more significant differences in the final epidemic size as ${\mathcal {R}}_{0}$ gets larger in the right panels.

**Fig. 2:**
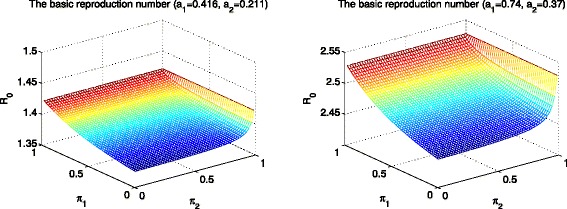
The impact of mixing patterns on the basic reproduction number $\mathcal {R}_{0}$. The basic reproduction number $\mathcal {R}_{0}$ is displayed as a function of *π*_1_ and *π*_2_, when activity levels are fixed (a moderate *R*_0_ in the *left panel* and a higher *R*_0_ in the *right panel*)

Next, the basic reproduction number ${\mathcal {R}}_{0}$ is displayed as a function of group mixing fractions in Fig. 2. The left panel shows the basic reproduction number $\mathcal {R}_{0}$ using a moderate value of activity levels ($\mathcal {R}_{0} \in \, [\!1.35, 1.45]$) while the right one using a higher value of activity levels ($\mathcal {R}_{0} \in \, [\!2.45, 2.55]$). Both panels show that the basic reproduction number gets slightly larger as preferred mixing becomes like-with-like mixing (either *π*_1_ or *π*_2_ becomes 1). This is consistent with the analytic expression for $\mathcal {R}_{0}$. Since *p*_11_ and *p*_22_ increase and *p*_12_ and *p*_21_ decrease as either *π*_1_ or *π*_2_ become 1, the basic reproduction number increases as preferred mixing becomes like-with-like mixing. Using the parameter values given here, it is worth mentioning that the effect of the lower activity group mixing fraction (*π*_2_) on the values of ${\mathcal {R}}_{0}$ is slightly more significant than the higher activity group mixing fraction (*π*_1_). The slope for the axis of *π*_2_ increases more than the slope for the axis of *π*_1_ in both panels.

**Fig. 3:**
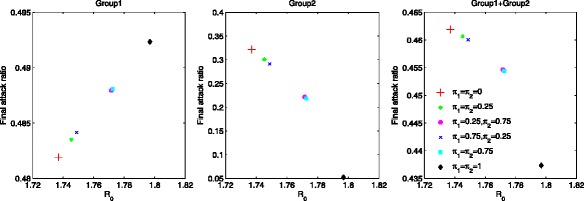
The impact of mixing patterns on the final attack ratio. The impact of different mixing patterns on the final attack ratio is displayed using *a*_1_=0.526 and *a*_2_=0.267. All results are in order of *π*_2_, whether decreasing or increasing and regardless of the different combinations of mixing fractions

We compute the final epidemic size, which is the number of members of the population who are infected over the course of the epidemic, *N*−*S*_*∞*_ with $S_{\infty }={\lim }_{t\rightarrow \infty } S(t)$. This can be described in terms of the final attack ratio, (1−*S*_*∞*_/*N*). In Fig. 3, the final attack ratio is displayed under various mixing scenarios (six different combinations of *π*_1_ and *π*_2_). The left and middle panels show the final attack ratios for group 1 and group 2, respectively, while the right panel shows the total final attack ratio. Note that the range of ${\mathcal {R}}_{0}$ is between 1.72 and 1.82 (*x*-axis) using *a*_1_=0.526 and *a*_2_=0.267 as the mixing fractions are varied. The final attack ratio for group 1 gets larger as the mixing becomes like-with-like mixing (*π*_1_=*π*_2_=1), while it becomes significantly smaller in group 2. Consequently, the total final attack ratio becomes smaller as the mixing becomes like-with-like mixing. Proportionate mixing makes the individuals in group 2 more likely to get infected than like-with-like mixing resulting in a significantly increased the final attack ratio in group 2. This leads to the result that the total final attack ratio follows exactly the same order (i.e. the total final attack ratio becomes smaller as *π*_2_ becomes to 1 in the right panel). Moreover, all results follow the order of a mixing fraction for the group 2 (*π*_2_) whether decreasing or increasing in the final attack ratio (all panels). Therefore, the basic reproduction number and the final attack ratio are not consistent and this reconfirms that the basic reproduction number alone is not sufficient to determine whether preferred mixing increases the final epidemic size or not [21].

These results are dependent on the group activity level, the group mixing fraction, and the group population size. We present a summary of the impact of these parameters on the final attack ratio as group activity levels and group population sizes are varied from the baseline scenarios in the next section.

### The impact of mixing patterns on the group-specific optimal treatment

**Fig. 4:**
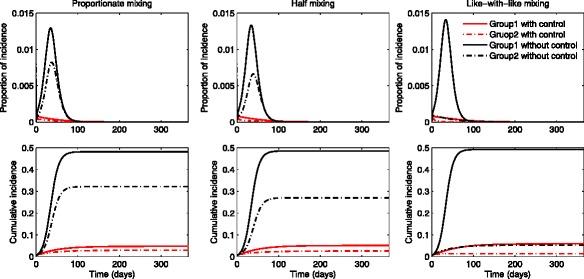
The impact of optimal age-specific controls under different mixing patterns. The proportion of group-specific incidence in the presence of optimal treatment (*red curves*) is displayed under the three mixing patterns for a moderate value of $\mathcal {R}_{0}$. The results are compared with the ones in the absence of treatment (*black curves*)

**Fig. 5:**
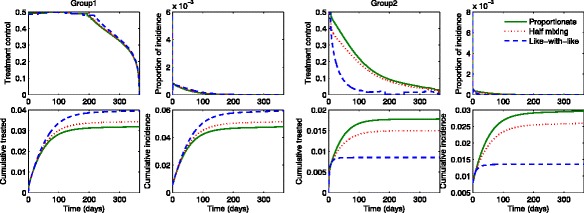
Optimal age-specific controls under different mixing patterns. Optimal group-specific treatment and the corresponding incidence are displayed (*top panels*) under the three mixing patterns using a moderate value of $\mathcal {R}_{0}$. The proportion of cumulative treated and cumulative infected individuals are displayed under proportionate mixing, half mixing and like-with-like mixing (*bottom panels*)

We present the numerical simulations associated with implementing optimal treatment control functions as well as their effect on two-group influenza dynamics under different mixing patterns. Also, the impact of different levels of transmissibility is investigated by varying the basic reproduction number. Figures 4 and 5 show the results under three different mixing patterns using a moderate value of *R*_0_∈ [ 1.73,1.79]. Likewise in the previous section, three mixing patterns are chosen as proportionate mixing, half mixing and like-with-like mixing. In Fig. 4, the proportion of incidence and cumulative incidence in the presence of optimal treatments (red curves) are compared with the results in the absence of treatments (black curves). The results show that there are no outbreak in the presence of treatments and this indicates that group-specific optimal treatments are effective enough to prevent outbreaks regardless of mixing patterns.

Figure 5 illustrates the impact of mixing patterns on the group-specific optimal treatment controls and the proportion of incidence under the three mixing patterns. Note that the time window of treatment is wider for group 1 under all mixing patterns while the time period of treatment becomes smaller for group 2 as the mixing becomes like-with-like (top panels). This is due to the fact that group 1 has a higher activity level and a larger population size than group 2. Also, the cumulative treated proportion becomes smaller as mixing becomes like-with-like for group 2, while it is the opposite for group 1. As noted in the previous section, the incidence in the lower activity level group 2 gets significantly larger as the mixing becomes more proportionate than the one in the higher activity group 1. Hence, this leads to the final epidemic size getting smaller and it requires less treatment as mixing becomes like-with-like mixing (bottom panels).

**Fig. 6:**
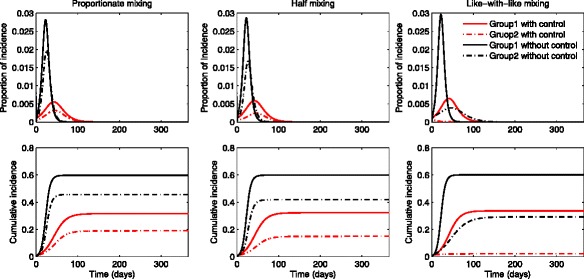
The impact of optimal age-specific controls under different mixing patterns. The proportion of group-specific incidence in the presence of optimal treatment (*red curves*) is displayed under the three mixing patterns for a higher value of $\mathcal {R}_{0}$. The results are compared with the ones in the absence of treatment (*black curves*)

**Fig. 7:**
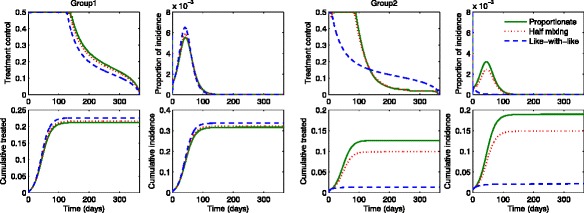
Optimal age-specific controls under different mixing patterns. Optimal group-specific treatment and the corresponding incidence are displayed (*top panels*) under the three mixing patterns using a higher value of $\mathcal {R}_{0}$. The proportion of cumulative treated and cumulative infected individuals are displayed under proportionate mixing, half mixing and like-with-like mixing (*bottom panels*)

Figures 6 and 7 show the results under three different mixing patterns using a higher value of $\mathcal {R}_{0} \in \, [\!2.45,2.53]$ and higher activity levels *a*_1_=0.742,*a*_2_=0.377. Again, Fig. 6 shows the proportion of incidence and cumulative in the presence of optimal treatments (red curves) are compared with the results in the absence of treatments (black curves). Since the basic reproduction number becomes higher, optimal treatments can not stop the outbreaks under all mixing patterns. Figure 7 displays the group-specific optimal treatment controls and the proportion of incidence. We observe that the time period of treatment gets smaller for group 1 but larger for group 2, than the ones using a moderate $\mathcal {R}_{0}$. Hence, this results in the cumulative treated cases increasing significantly in both groups. As $\mathcal {R}_{0}$ becomes higher, the number of infected individuals in group 2 increases dramatically as the mixing becomes proportionate. Note that group 2 (the lower activity group) is more sensitive to mixing patterns.

### The impact of mixing patterns on the group-specific final epidemic size

**Fig. 8:**
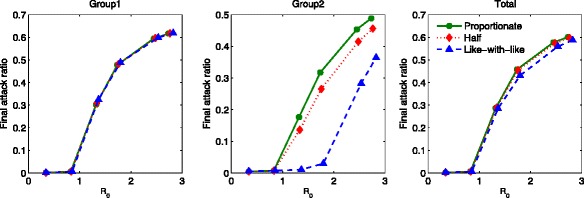
Final attack ratio in the absence of treatment. The final attack ratio in the absence of treatment is displayed as a function of $\mathcal {R}_{0}$ under three mixing patterns. The basic reproduction number is increased as the activity levels increase in the range of *a*_1_∈ [ 0.1,0.827] and *a*_2_∈ [ 0.05,0.42]

Figure 8 displays the group-specific final attack ratio and the total final attack ratio as a function of ${\mathcal {R}}_{0}$ in the absence of treatment under three distinct mixing patterns. The basic reproduction number is increased as the activity levels are increased in the ranges of *a*_1_∈ [ 0.1,0.827] and *a*_2_∈ [ 0.05,0.42]. The left panel shows that the final attack ratio for group 1 becomes almost indistinguishable regardless of mixing. The final attack ratio for group 2 has the largest value under proportionate mixing (circled), while it becomes smaller as the mixing becomes like-with-like (triangle). This results in the fact that the total (both groups) final attack ratio follows the order of group 2.

**Fig. 9:**
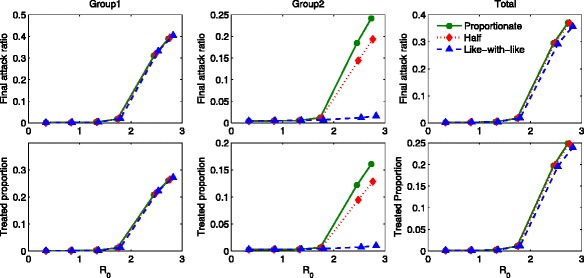
Final attack ratio in the presence of treatment. The impact of the mixing patterns on the final attack ratio is illustrated. The final attack ratio in the presence of optimal treatment strategies is displayed as a function of $\mathcal {R}_{0}$ under the three mixing patterns

Figure 9 presents the comparisons of the final attack ratio and the proportion of cumulative treated as a function of ${\mathcal {R}}_{0}$ in the presence of treatment. It is clear how optimal treatment strategies and the mixing fractions affect the final attack ratio. Similar to the results in the absence of treatment, the final attack ratio for group 1 is almost indistinguishable under all mixing patterns. Again, the final attack ratio for group 2 becomes smaller as the mixing becomes like-with-like. However, these results show that optimal treatment strategies can significantly limit the severity of outbreaks when ${\mathcal {R}}_{0}$ is brought below a certain threshold (the controlled reproduction number, ${\mathcal {R}}_{c}$). Particularly, for the lower activity group, the reduction is dramatic (triangle in the top middle panel). The cumulative treated results are consistent with the final attack ratio results (more infected, more treatment needed in the bottom panels).

### The impact of control parameters

**Fig. 10:**
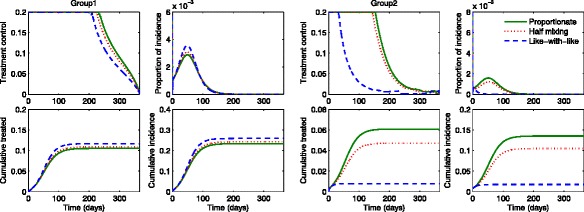
The impact of different control upper bounds. The optimal group-specific treatment and the corresponding incidence are displayed under the three mixing patterns. The results are presented using a lower control bound (*b*_1_=*b*_2_=0.2)

There are two critical control parameters that change the corresponding dynamics greatly. One of them is the control upper bound, *b*_*i*_, which represents the maximum level of effectiveness for implementing the treatment. The influenza outcomes are dependent on the control upper bound in a straightforward fashion. As the control upper bound is decreased, the magnitude of control decreases for all mixing patterns as shown in Fig. 10. This leads to a longer time of treatment in both groups and interestingly, it results in larger costs (more treated cases) and larger infected cases. This indicates that the higher treatment rate is more effective (less treated cases and less infected as seen in Fig. 5).

**Fig. 11:**
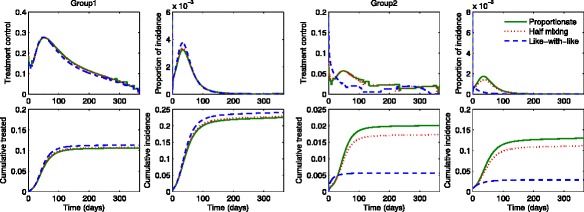
The impact of different weight constants. The optimal group-specific treatment and the corresponding incidence are displayed under the the three mixing patterns. The results are presented using a higher weight constant (*W*_1_=*W*_2_=100)

The other control parameters are the weight constants or the relative costs of treatments which can play a critical role in the influenza dynamics. As we increase these parameters, *the relative cost* of control increases and therefore, the magnitude of the optimal controls decreases, resulting in the increase of infected individuals and the final epidemic size in both groups regardless of the mixing pattern in Fig. 11.

### The impact of different activity levels and subpopulation sizes

All simulation results so far have been based on the case where group 1 is a higher activity group and a larger population size than the ones for group 2 (*a*_1_>*a*_2_, *N*_1_>*N*_2_). Now we investigate the impact of different group activity levels and subpopulation sizes on the optimal treatments and the resulting two group influenza dynamics. There are a total of nine scenarios as we vary group activity levels (*a*_1_,*a*_2_) and subpopulation sizes (*N*_1_,*N*_2_). Only some selected results are presented because the rest of the cases are identical.

- Baseline scenario: *a*_1_>*a*_2_, *N*_1_>*N*_2_
- Scenario 1: *a*_1_>*a*_2_, *N*_1_=*N*_2_
- Scenario 2: *a*_1_>*a*_2_, *N*_1_<*N*_2_
- Scenario 3: *a*_1_=*a*_2_, *N*_1_=*N*_2_
- Scenario 4: *a*_1_<*a*_2_, *N*_1_<*N*_2_

1. Let us consider the first scenario for *a*_1_>*a*_2_ and *N*_1_=*N*_2_: the number of infected and treated cases for group 1 under the baseline scenario is larger than the ones under the first scenario, while it is the opposite for group 2 under all mixing patterns. However, the total number of infected and treated cases is larger under the baseline scenario than the first scenario. When mixing is proportionate (*π*_*i*_=0), the total cumulative treated (infected) number of the baseline scenario is 403 (894) and the one for the first scenario is 376 (837), respectively. Now, when the mixing is like-with-like (*π*_*i*_=1), the total cumulative treated number of the baseline scenario is 399 (885) and the one for the first scenario is 236 (522), respectively. Interestingly, the baseline scenario requires more treatment, but the number of infected people is larger than the first scenario, and the difference becomes more significant as mixing becomes more like-with-like.

2. The second scenario is *a*_1_>*a*_2_ and *N*_1_<*N*_2_: this scenario is for the case where the higher activity group has a smaller population size than the lower activity group. The total cumulative treated (infected) number is 360 (799) for proportionated mixing and 73 (159) for like-with-like mixing, respectively. This result demonstrates that optimal treatment has more significant impact to reduce the epidemic size as mixing become like-with-like when a higher activity group has a smaller subpopulation size. The time using treatment control for group 2 is longer than the one of group 1 under proportionate mixing. However, the time using treatment of group 2 decreases by increasing *π*_1_, so that the time of treatment for group 1 is longer than the one for group 2 as mixing becomes like-with-like. This suggests that the time duration of implementing treatment depends on the group activity levels (the higher activity requires a longer time to implement).

3. The third scenario is that the activity level and the population size for group 1 and group 2 are the same. The basic reproduction number, the final attack ratios and optimal group-specific treatments are almost the same under all mixing patterns. It shows that the impact of mixing patterns is not significant when groups have the same activity level and the same population size. Lastly, the scenario 4 for *a*_1_<*a*_2_ and *N*_1_<*N*_2_ is exactly identical as the baseline scenario, hence, the results are omitted.

## Conclusions

We have studied the dynamics of influenza transmission in a two-group model in which two groups are connected via a mixing matrix. The model proposed here represents two age groups with different activity levels and distinct mixing patterns. Several mixing patterns are considered such as proportionate and preferred mixing by varying the group mixing fractions *π*_*i*_ for *i*=1,2. The impact of these mixing patterns is illustrated on the basic reproduction number and the group specific final epidemic size. Also, the intensity of ${\mathcal {R}}_{0}$ is varied by using different values of the group specific activity levels.

The basic reproduction number ${\mathcal {R}}_{0}$ increases as the mixing becomes like-with-like mixing. Interestingly, in the absence of treatments, the opposite is true for the final epidemic size, which gets smaller as mixing becomes like-with-like as reported [23]. This is consistent with the observations that the basic reproduction number alone is not enough to determine the final epidemic size in a heterogeneous model [21]. However, the basic reproduction number and the final epidemic size depend on the group activity level and the group population size as well. Under our baseline scenarios (*a*_1_>*a*_2_ and *N*_1_>*N*_2_), the final attack ratios decreases as *π*_2_ increases to 1, which implies that the group 2 mixing fraction determines the order of the final attack ratio. Using different sets of parameters, the order might changes depending on either *π*_1_ or *π*_2_.

Furthermore, we formulated an optimal framework to investigate group-specific optimal treatment strategies under various mixing scenarios. For a moderate value of $\mathcal {R}_{0}$, the optimal treatment can prevent the outbreaks in both groups under all mixing patterns. The treatment time is longer for group 1 and the treated cases are larger since it has a higher activity level and a large population size, regardless of mixing patterns. For a higher value of $\mathcal {R}_{0}$, the optimal treatment can not stop the outbreaks in both groups under all mixing patterns. Compared with the moderate $\mathcal {R}_{0}$ results, the treatment time for group 1 decreases, but for group 2 increases. This is due to the fact that the number of those infected in the lower activity group gets significantly larger as the mixing becomes more proportionate. Therefore, treating more people is necessary with emphasis on the lower activity group, when two groups mix proportionately.

Optimal treatment strategies can significantly limit the severity of outbreaks when ${\mathcal {R}}_{0}$ is brought below a certain threshold (the controlled reproduction number, ${\mathcal {R}}_{c}$). Under optimal treatments in both groups, the controlled reproduction number ${\mathcal {R}}_{c}$ and the final attack ratio decrease slightly as mixing becomes like-with-like. Again, the final attack ratio is in the order of either *π*_1_ or *π*_2_. Preferred mixing changes the basic reproduction number, the controlled reproduction number and the final epidemic size in a rather complex way and the effect gets more substantial as the epidemic gets more severe.

Further, the impact of different group activity levels and subpopulation sizes are explored for the optimal treatments and the resulting two group influenza dynamics. First, the group mixing fractions can play a key role in the final attack ratio. For the case of *a*_1_>*a*_2_ and *N*_1_=*N*_2_, *π*_2_ determines the order of the final attack ratio, i.e., the total final attack ratio becomes smaller as *π*_2_ becomes 1. For the case of *a*_1_>*a*_2_ and *N*_1_<*N*_2_, the total final attack ratio becomes smaller as *π*_1_ becomes 1. Based on these results, the effectiveness of optimal treatments is dependent on the group specific parameters. In general, the optimal treatment becomes more efficient as the mixing becomes more like-with-like. The efficiency of optimal treatments becomes more substantial when a higher activity group has a smaller population size (*a*_1_>*a*_2_ and *N*_1_<*N*_2_). Also, the time duration of implementing treatment depends on the group activity levels while the final attack ratios are more sensitive to the group population size.

Sensitivity analysis for the control upper bound and the weight constant has been carried out. The results using a lower upper bound (*b*=0.2) and a higher weight constant (*W*=100) show that the magnitude of the treatment controls decreases and therefore, the total amount of treated and infected cases are increased in both groups under all mixing patterns. Clearly, this indicates that a more intensive treatment or a higher treatment rate is able to more efficiently reduce the total number of infected individuals with less treatment.

For the parameters used here, our results indicate that treatment of both groups with a higher rate is the most effective, regardless of mixing scenarios. However, proportionate mixing requires more treated cases for all combinations of different group activity levels and group population sizes. In other word, as the mixing becomes more like-with-like mixing, treatment of the more active group in the population is almost as effective as treating the entire population, since it reduces the number of disease cases effectively but requires the similar treatments. The gain is more pronounced as the basic reproduction number increases. This can be a critical issue which has to be considered for future epidemic interventions, especially when there are limited resources.

This study focuses on the two-group influenza model to explore the effect of heterogeneous mixing on the group-specific optimal treatment. This simple two-group influenza model can be used for any general disease which consists of two different activity levels and different mixing patterns. Furthermore, this work can be generalized to a multi-group influenza model (with more age groups) so that it can capture more interesting and realistic epidemiological scenarios. This will be carried out in our future study.

## Appendix A

The basic reproductive number is calculated by using the methodology (the next generation matrix approach) outlined in [26]. Now, let *F*(**x**) represent the rate of appearance of new infections. The net transition rates out of the corresponding compartment are represented by *V*(**x**). Then, we find the Jacobian matrix of *F*(**x**) and *V*(**x**) and denote them ${\mathbf {F}}=\left [\frac {\partial F}{\partial \mathbf {x}_{j}}\right ]$ and $\textbf {V}=\left [\frac {\partial V}{\partial \mathbf {x}_{j}}\right ]$, evaluated at the disease free equilibrium point **x**^∗^, which consists of *S*_1_=*N*_1_, *S*_2_=*N*_2_ with the rest of them zero.

$$ \mathbf{F} = \left[ \begin{array}{cccccccc} 0 & a_{1} p_{11} \frac{S_{1}}{N_{1}} & a_{1} p_{11} \frac{S_{1} \delta}{N_{1}} & a_{1} p_{11}\frac{S_{1} \sigma}{N_{1}} & 0 & a_{1} p_{12} \frac{S_{1}}{N_{2}} & a_{1} p_{12} \frac{S_{1} \delta}{N_{2}} & a_{1} p_{12}\frac{S_{1} \sigma}{N_{2}}\\ 0 & 0 & 0 & 0 & 0 & 0 & 0 & 0 \\ 0 & 0 & 0 & 0 & 0 & 0 & 0 & 0 \\ 0 & 0 & 0 & 0 & 0 & 0 & 0 & 0 \\ 0 & a_{2} p_{21} \frac{S_{2}}{N_{1}} & a_{2} p_{21} \frac{S_{2} \delta}{N_{1}} & a_{2} p_{21}\frac{S_{2} \sigma}{N_{1}} & 0 & a_{2} p_{22} \frac{S_{2}}{N_{2}} & a_{2} p_{22} \frac{S_{2} \delta}{N_{2}} & a_{2} p_{22}\frac{S_{2} \sigma}{N_{2}}\\ 0 & 0 & 0 & 0 & 0 & 0 & 0 & 0 \\ 0 & 0 & 0 & 0 & 0 & 0 & 0 & 0 \\ 0 & 0 & 0 & 0 & 0 & 0 & 0 & 0 \\ \end{array} \right]. $$

In **F**, $a_{1}p_{12}\frac {S_{1}}{N_{2}}$ is replaced by the balance relation $\frac {a_{2} p_{1}}{N_{1}}=\frac {a_{1} p_{2}}{N_{2}}$. Then, we get

$$ a_{1}((1-\pi_{1})p_{2})\frac{S_{1}}{N_{2}}=a_{2}((1-\pi_{1})p_{1})\frac{S_{1}}{N_{1}}. $$

Also,

$$\mathbf{V} = \left[ \begin{array}{cccccccc} \kappa_{1} & 0 & 0 & 0 & 0 & 0 & 0 & 0\\ -p\kappa_{1} & \alpha_{1}+u_{1} & 0 & 0 & 0 & 0 & 0 & 0\\ -(1-p)\kappa_{1} & 0 & \eta_{1} & 0 & 0 & 0 & 0 & 0\\ 0 & -u_{1} & 0 & \alpha_{T,1} & 0 & 0 & 0 & 0\\ 0 & 0 & 0 & 0 & \kappa_{2} & 0 & 0 & 0\\ 0 & 0 & 0 & 0 & -p\kappa_{2} & \alpha_{2}+u_{2} & 0 & 0\\ 0 & 0 & 0 & 0 & -(1-p)\kappa_{2} & 0 & \eta_{2} & 0\\ 0 & 0 & 0 & 0 & 0 & -u_{2} & 0 & \alpha_{T,2} \end{array} \right]. $$

The matrix **FV**^−1^ has six zero eigenvalues and the remaining two eigenvalues are the roots of the following quadratic equation:

$$\lambda^{2}-(p_{11}a_{1}\Gamma_{1}+p_{22}a_{2}\Gamma_{2})\lambda+(p_{11}p_{22}-p_{12}p_{21})a_{1}a_{2}\Gamma_{1}\Gamma_{2}=0. $$

The controlled reproduction $\mathcal {R}_{c}$ is the largest of these two eigenvalues, which is

(8) $$ \mathcal{R}_{c}=\frac{1}{2}\left(a_{1}p_{11}\Gamma_{1}+a_{2}p_{22}\Gamma_{2}+\sqrt{(a_{1}p_{11} \Gamma_{1}-a_{2}p_{22}\Gamma_{2})^{2}+4a_{1}a_{2}p_{12}p_{21}\Gamma_{1}\Gamma_{2}} \right),  $$

where $\Gamma _{1}=\left (\frac {\delta (1-p)}{\eta _{1}}+\frac {p(\alpha _{T,1}+\sigma u_{1})}{\alpha _{T,1}(\alpha _{1}+u_{1})} \right), \Gamma _{2}=\left (\frac {\delta (1-p)}{\eta _{2}}+\frac {p(\alpha _{T,2}+\sigma u_{2})}{\alpha _{T,2}(\alpha _{2}+u_{2})} \right)$.

The basic reproduction number $\mathcal {R}_{0}$ is $\mathcal {R}_{c}$ with *u*_1_=*u*_2_=0:

$$\mathcal{R}_{0}=\frac{1}{2}\left(a_{1}p_{11}\Phi_{1}+a_{2}p_{22}\Phi_{2}+\sqrt{(a_{1}p_{11}\Phi_{1}-a_{2}p_{22}\Phi_{2})^{2}+4a_{1}a_{2}p_{12}p_{21}\Phi_{1}\Phi_{2}} \right), $$

where $\Phi _{1}=\left (\frac {\delta (1-p)}{\eta _{1}}+\frac {p}{\alpha _{1}} \right), \Phi _{2}=\left (\frac {\delta (1-p)}{\eta _{2}}+\frac {p}{\alpha _{2}} \right)$.

Next, we compute the final size relation by introducing the notation *g*(*∞*) for ${\lim }_{\textit {t}\rightarrow \infty }g(t)$ and $\hat {g}$ for $\int _{0}^{\infty }g(t)dt$ assuming *g* is a nonnegative integrable function defined for 0≤*t*<*∞*.

$$\begin{aligned} &L_{1}(\infty)=0, \ \ I_{1}(\infty)=0, \ \ A_{1}(\infty)=0, \ \ T_{1}(\infty)=0,\\ &L_{2}(\infty)=0, \ \ I_{2}(\infty)=0, \ \ A_{2}(\infty)=0, \ \ T_{2}(\infty)=0,\\ &S_{1}(0)+L_{1}(0)-S_{1}(\infty)=N_{1}(0)-S_{1}(\infty)=\kappa_{1}L_{1},\\ &S_{2}(0)+L_{2}(0)-S_{2}(\infty)=N_{2}(0)-S_{2}(\infty)=\kappa_{2}L_{2}. \end{aligned} $$

Also, we have the following:

$$\begin{aligned} \phi_{1}\hat{I_{1}}&=\alpha_{T,1}\hat{T_{1}}, \ \ \phi_{2}\hat{I_{2}}=\alpha_{T,2}\hat{T_{2}},\\ (1-\rho)\kappa_{1}\hat{L_{1}}&=\eta_{1}\hat{A_{1}}, \ \ (1-\rho)\kappa_{2}\hat{L_{2}}=\eta_{2}\hat{A_{2}}, \\ \rho\kappa_{1}\hat{L_{1}}&=(\alpha_{1}+u_{1})\hat{I_{1}}, \ \ \rho\kappa_{2}\hat{L_{2}}=(\alpha_{2}+u_{2})\hat{I_{2}}. \end{aligned} $$

Using the first equation in (1), we have

(9) $$ \begin{array}{lcl} -\frac{S_{1}'}{S_{1}}=\left[\frac{a_{1} p_{11}}{N_{1}}(I_{1}+\sigma T_{1}+\delta A_{1})+\frac{a_{1} p_{12}}{N_{2}}(I_{2}+\sigma T_{2}+\delta A_{2})\right]. \end{array}  $$

Integrating Eq. (9),

(10) $$ \begin{aligned} \ln\frac{S_{1}(0)}{S_{1}(\infty)}&=\frac{a_{1}p_{11}}{N_{1}}\left(\hat{I_{1}}+\sigma \hat{T_{1}}+\delta\hat{A_{1}}\right)+\frac{a_{1} p_{12}}{N_{2}}\left(\hat{I_{2}}+\hat{\sigma T_{2}}+\hat{\delta A_{2}}\right)\\ &= a_{1} p_{11}\left(\frac{\rho(\alpha_{T,1}+\sigma u_{1})}{\alpha_{T,1}(\alpha_{1}+ u_{1})}+\frac{(1-\rho)\delta}{\eta_{1}}\right)\left(1-\frac{S_{1}(\infty)}{N_{1}} \right)\\ &\quad+a_{1}p_{12}\left(\frac{\rho(\alpha_{T,2}+\sigma u_{2})}{\alpha_{T,2}(\alpha_{2}+ u_{2})}+\frac{(1-\rho)\delta}{\eta_{2}}\right)\left(1-\frac{S_{2}(\infty)}{N_{2}}\right)\\ \therefore \ln\frac{S_{1}(0)}{S_{1}(\infty)}&= a_{1} p_{11}\Gamma_{1}\left(1-\frac{S_{1}(\infty)}{N_{1}} \right)+a_{1} p_{12}\Gamma_{2}\left(1-\frac{S_{2}(\infty)}{N_{2}}\right). \end{aligned}  $$

Similarly,

(11) $$ \begin{aligned} -\frac{S_{2}'}{S_{2}}=\left[ \frac{a_{2} p_{21}}{N_{1}}(I_{1}+\sigma T_{1}+\delta A_{1})+\frac{a_{2} p_{22}}{N_{2}}(I_{2}+\sigma T_{2}+\delta A_{2})\right]. \end{aligned}  $$

Also, integrating Eq. (11),

(12) $$ \begin{aligned} \ln\frac{S_{2}(0)}{S_{2}(\infty)}&=\frac{a_{2} p_{21}}{N_{1}}(\hat{I_{1}}+\hat{\sigma T_{1}}+\hat{\delta A_{1}})+\frac{a_{2} p_{22}}{N_{2}}\left(\hat{I_{2}}+\hat{\sigma T_{2}}+\hat{\delta A_{2}}\right)\\ &= a_{2} p_{21}\left(\frac{\rho(\alpha_{T,1}+\sigma u_{1})}{\alpha_{T,1}(\alpha_{1}+ u_{1})}+\frac{(1-\rho)\delta}{\eta_{1}}\right)\left(1-\frac{S_{1}(\infty)}{N_{1}} \right)\\ &\quad +a_{2}p_{22}\left(\frac{\rho(\alpha_{T,2}+\sigma u_{2})}{\alpha_{T,2}(\alpha_{2}+ u_{2})}+\frac{(1-\rho)\delta}{\eta_{2}}\right)\left(1-\frac{S_{2}(\infty)}{N_{2}}\right),\\ \therefore \ln\frac{S_{2}(0)}{S_{2}(\infty)}&= a_{2} p_{21}\Gamma_{1}\left(1-\frac{S_{1}(\infty)}{N_{1}} \right)+a_{2} p_{22}\Gamma_{2}\left(1-\frac{S_{2}(\infty)}{N_{2}}\right). \end{aligned}  $$

The final size relation in the absence of treatment (*u*_1_=*u*_2_=0) can be written as:

(13) $$ \left[ \begin{array}{c} \ln\frac{S_{1}(0)}{S_{1}(\infty)} \\ \ln\frac{S_{2}(0)}{S_{2}(\infty)} \end{array} \right] = \left[ \begin{array}{c} a_{1} p_{11}\Phi_{1}\left(1-\frac{S_{1}(\infty)}{N_{1}(0)} \right)+a_{1} p_{12}\Phi_{2}\left(1-\frac{S_{2}(\infty)}{N_{2}(0)}\right) \\ a_{2} p_{21}\Phi_{1}\left(1-\frac{S_{1}(\infty)}{N_{1}(0)} \right)+a_{2} p_{22}\Phi_{2}\left(1-\frac{S_{2}(\infty)}{N_{2}(0)}\right) \end{array} \right].  $$

## Appendix B

The optimal control problem for the two-group influenza model is formulated to minimize the number of infected individuals for a finite time interval at a minimal cost. We define our objective functional as follows:

(14) $$ \mathcal{F}\left(u_{1}(t),u_{2}(t)\right) ={\int_{0}^{T}} \left(C_{1}I_{1}(t)+C_{2}I_{2}(t)+\frac{W_{1}}{2}{u_{1}^{2}}(t)+\frac{W_{2}}{2}{u_{2}^{2}}(t) \right) dt.  $$

Then, we seek an optimal pair (*U*^∗^,*X*^∗^) such that

(15) $$ \mathcal{F}(u_{1}^{*}(t), u_{2}^{*}(t)) = min_{\Omega} \mathcal {F}(u_{1}(t),u_{2}(t)),  $$

where *Ω*={(*u*_1_(*t*),*u*_2_(*t*))∈(*L*^1^(0,*T*))^2^∥0≤*u*_1_(*t*),*u*_2_(*t*)≤*b, t*∈ [ 0,*T*]} subject to the state equations given by (1) with initial conditions. The existence of optimal controls is guaranteed from standard results in optimal control theory [36]. The necessity conditions of optimal solutions are derived from Pontryagin’s Maximum Principle [37]. This principle converts the systems (1), (6), (7) into the problem of minimizing the Hamiltonian *H* given by

(16) $$ \begin{aligned} H&= C_{1}I_{1}(t)+C_{2}I_{2}(t)+\frac{W_{1}}{2}{u_{1}^{2}}(t)+\frac{W_{2}}{2}{u_{2}^{2}}(t)\\ &\quad +\lambda_{S_{1}}\left(-a_{1}\left[p_{11}\frac{S_{1}(I_{1}+\sigma T_{1}+\delta A_{1})}{N_{1}}+p_{12}\frac{S_{1}(I_{2}+\sigma T_{2}+\delta A_{2})}{N_{2}}\right]\right)\\ &\quad +\lambda_{S_{2}}\left(-a_{2}\left[p_{21}\frac{S_{2}(I_{1}+\sigma T_{1}+\delta A_{1})}{N_{1}}+p_{22}\frac{S_{2}(I_{2}+\sigma T_{2}+\delta A_{2})}{N_{2}}\right]\right)\\ &\quad +\lambda_{L_{1}}\left(a_{1}\left[p_{11}\frac{S_{1}(I_{1}+\sigma T_{1}+\delta A_{1})}{N_{1}}+p_{12}\frac{S_{1}(I_{2}+\sigma T_{2}+\delta A_{2})}{N_{2}}\right]-\kappa_{1}L_{1}\right)\\ &\quad +\lambda_{L_{2}}\left(a_{2}\left[p_{21}\frac{S_{2}(I_{1}+\sigma T_{1}+\delta A_{1})}{N_{1}}+p_{22}\frac{S_{2}(I_{2}+\sigma T_{2}+\delta A_{2})}{N_{2}}\right] -\kappa_{2}L_{2}\right)\\ &\quad +\lambda_{I_{1}}\left(p\kappa_{1}L_{1}-(\alpha_{1}+u_{1}(t))I_{1}\right) +\lambda_{I_{2}}\left(p\kappa_{2}L_{2}-(\alpha_{2}+u_{2}(t))I_{2}\right)\\ &\quad +\lambda_{A_{1}}\left((1-p)\kappa_{1}L_{1}-\eta_{1}A_{1}\right) +\lambda_{A_{2}}\left((1-p)\kappa_{2}L_{2}-\eta_{2}A_{2}\right)\\ &\quad +\lambda_{T_{1}}\left(u_{1}(t)I_{1}-\alpha_{T,1}T_{1}\right) +\lambda_{T_{2}}\left(u_{2}(t)I_{2}-\alpha_{T,2}T_{2}\right) \end{aligned}  $$

From this Hamiltonian and Pontryagin’s Maximum Principle [37], we obtain the following theorem:

### **Theorem**.

There exist optimal controls $u_{1}^{*}(t), u_{2}^{*}(t)$ and corresponding solutions, $S^{*}_{i}, L^{*}_{i}$, $I^{*}_{i}$, $A^{*}_{i}$ and $T^{*}_{i}$ that minimizes $\mathcal {F}(u_{1}(t), u_{2}(t))$ over the domain *Ω*. In order for the above statement to be true, it is necessary that there exist continuous functions $\lambda _{S_{i}(t)}$, $\lambda _{L_{i}(t)}$, $\lambda _{I_{i}(t)}$, $\lambda _{A_{i}(t)}$ and $\lambda _{T_{i}(t)}$ for *i*=1,2 such that

(17) $$ \begin{aligned} \dot \lambda_{S_{i}(t)}&= a_{i}\left\{ p_{i1}\frac{(I_{1}+\sigma T_{1}+\delta A_{1})}{N_{1}}+p_{i2}\frac{(I_{2}+\sigma T_{2}+\delta A_{2})}{N_{2}}\right\} (\lambda_{S_{i}}-\lambda_{L_{i}}),\\ \dot \lambda_{L_{i}(t)}&= \kappa_{i}\left\{ (\lambda_{L_{i}}-\lambda_{A_{i}})-p(\lambda_{I_{i}}-\lambda_{A_{i}}) \right\},\\ \dot\lambda_{I_{i}(t)}&= -C_{i}+a_{1}p_{1i}\frac{S_{1}}{N_{i}}(\lambda_{S_{1}}-\lambda_{L_{1}})+a_{2}p_{2i} \frac{S_{2}}{N_{i}}(\lambda_{S_{2}}-\lambda_{L_{2}})+(\alpha_{i}+u_{i})\lambda_{I_{i}}-u_{i}\lambda_{T_{i}},\\ \dot \lambda_{A_{i}(t)}&= a_{1}p_{1i}\frac{S_{1}\delta}{N_{i}}(\lambda_{S_{1}}-\lambda_{L_{1}})+a_{2}p_{2i} \frac{S_{2}\delta}{N_{i}}(\lambda_{S_{2}}-\lambda_{L_{2}})+\eta_{i} \lambda_{A_{i}},\\ \dot \lambda_{T_{i}(t)}&= a_{1}p_{1i}\frac{S_{1}\sigma}{N_{i}}(\lambda_{S_{1}}-\lambda_{L_{1}})+a_{2}p_{2i} \frac{S_{2}\sigma}{N_{i}}(\lambda_{S_{2}}-\lambda_{L_{2}})+\alpha_{T,i}\lambda_{T_{i}}, \end{aligned}  $$

with the transversality conditions,

$$ \lambda_{S_{i}}(T)=\lambda_{L_{i}}(T)=\lambda_{I_{i}}(T)=\lambda_{A_{i}}(T)=\lambda_{T_{i}}(T)=0, \text{ } i = 1,2. $$

Furthermore,

(18) $$ \begin{aligned} u_{1}^{*}(t) &= min\left\{ max\left\{0, \frac{I_{1}}{W1}(\lambda_{I_{1}} - \lambda_{T_{1}})\right\}, 1 \right\},\\ u_{2}^{*}(t) &= min\left\{ max\left\{0, \frac{I_{2}}{W2}(\lambda_{I_{2}} - \lambda_{T_{2}})\right\}, 1 \right\}. \end{aligned}  $$

### *Proof*.

The existence of optimal controls follows from Corollary 4.1 of [36] since the integrand of *J* is a convex function of *U*(*t*) and the state system satisfies the *Lipschitz* property with respect to the state variables. The following can be derived from the Pontryagin’s Maximum Principle [37]:

$$\begin{aligned} \lambda_{S_{1}}'=-\frac{\partial H}{\partial S_{1}} & =\left(\lambda_{S_{1}}-\lambda_{L_{1}}\right) \left[a_{1}\left(p_{11}\frac{(I_{1}+\sigma T_{1}+\delta A_{1})}{N_{1}}+p_{12}\frac{(I_{2}+\sigma T_{2}+\delta A_{2})}{N_{2}}\right)\right],\\ \lambda_{L_{1}}'=-\frac{\partial H}{\partial L_{1}} &=\kappa_{1}\left(\lambda_{L_{1}}-\lambda_{A_{1}}\right) +p\kappa_{1}\left(\lambda_{A_{1}}-\lambda_{I_{1}}\right),\\ \lambda_{I_{1}}'=-\frac{\partial H}{\partial I_{1}} &=-C_{1}+\left(\lambda_{S_{1}}-\lambda_{L_{1}}\right) \left(a_{1}p_{11}\frac{S_{1}}{N_{1}}\right) +\left(\lambda_{S_{2}}-\lambda_{L_{2}}\right) \left(a_{2}p_{21}\frac{S_{2}}{N_{1}}\right)\\ &\quad+\lambda_{I_{1}}\left(\alpha_{1}+u_{1}\right)-\lambda_{T_{1}}u_{1}, \end{aligned} $$

$$\begin{aligned} \lambda_{A_{1}}'=-\frac{\partial H}{\partial A_{1}} & =(\lambda_{S_{1}}-\lambda_{L_{1}})\left(a_{1}p_{11}\frac{\delta S_{1}}{N_{1}}\right) +(\lambda_{S_{2}}-\lambda_{L_{2}}) \left(a_{2}p_{21}\frac{\delta S_{2}}{N_{1}}\right)+\lambda_{A_{1}}\eta_{1}, \end{aligned} $$

$$\begin{aligned} \lambda_{T_{1}}'=-\frac{\partial H}{\partial T_{1}} & =(\lambda_{S_{1}}-\lambda_{L_{1}}) \left(a_{1}p_{11}\frac{\sigma S_{1}}{N_{1}}\right) +(\lambda_{S_{2}}-\lambda_{L_{2}})\left(a_{2}p_{21}\frac{\sigma S_{2}}{N_{1}}\right)+\lambda_{T_{1}}\alpha_{T,1}, \end{aligned} $$

$$\begin{aligned} \lambda_{S_{2}}'=-\frac{\partial H}{\partial S_{2}} &=\left(\lambda_{S_{2}}-\lambda_{L_{2}}\right) \left[a_{2}\left(p_{21}\frac{I_{1}+\sigma T_{1}+\delta A_{1}}{N_{1}}+p_{22}\frac{I_{2}+\sigma T_{2}+\delta A_{2}}{N_{2}}\right)\right], \\ \lambda_{L_{2}}'=-\frac{\partial H}{\partial L_{2}} & =\kappa_{2}\left(\lambda_{L_{2}}-\lambda_{A_{2}}\right)+p\kappa_{2}(\lambda_{A_{2}}-\lambda_{I_{2}}), \\ \lambda_{I_{2}}'=-\frac{\partial H}{\partial I_{2}} & =-C_{2}+(\lambda_{S_{1}}-\lambda_{L_{1}})\left(a_{1}p_{12}\frac{S_{1}}{N_{2}}\right) +(\lambda_{S_{2}}-\lambda_{L_{2}})\left(a_{2}p_{22}\frac{S_{2}}{N_{2}}\right)\\ &\quad +\lambda_{I_{2}}(\alpha_{2}+u_{2})-\lambda_{T_{2}}u_{2}, \end{aligned} $$

$$\begin{aligned} \lambda_{A_{2}}'=-\frac{\partial H}{\partial A_{2}} &=(\lambda_{S_{1}}-\lambda_{L_{1}}) \left(a_{1}p_{12}\frac{\delta S_{1}}{N_{2}}\right) +(\lambda_{S_{2}}-\lambda_{L_{2}}) \left(a_{2}p_{22}\frac{\delta S_{2}}{N_{2}}\right)+\lambda_{A_{2}}\eta_{2}, \end{aligned} $$

$$\begin{aligned} \lambda_{T_{2}}'=-\frac{\partial H}{\partial T_{2}} &=(\lambda_{S_{1}}-\lambda_{L_{1}}) \left(a_{1}p_{12}\frac{\sigma S_{1}}{N_{2}}\right) +(\lambda_{S_{2}}-\lambda_{L_{2}}) \left(a_{2}p_{22}\frac{\sigma S_{2}}{N_{2}}\right)+\lambda_{T_{2}}\alpha_{T,2}, \end{aligned} $$

with $\lambda _{S_{i}}$, $\lambda _{L_{i}}$, $\lambda _{I_{i}}$, $\lambda _{A_{i}}$, $\lambda _{T_{i}} (i=1,2)$ and evaluated at the optimal controls and corresponding states, which results in the adjoint system (17). The Hamiltonian *H* is minimized with respect to the controls at the optimal controls, so we differentiate *H* with respect to *u*_1_ and *u*_2_ on the set *Ω*, giving the following optimality conditions:

$$\begin{aligned} \frac{\partial H}{\partial u_{1}} = W_{1}u_{1}-\lambda_{I_{1}}I_{1}+\lambda_{T_{1}}I_{1} = 0,\\ \frac{\partial H}{\partial u_{2}} = W_{2}u_{2}-\lambda_{I_{2}}I_{2}+\lambda_{T_{2}}I_{2} = 0.\\ \end{aligned} $$

Solving for *u*_1_^∗^,*u*_2_^∗^, then

$$\therefore u_{1}^{*}=\frac{I_{1}(\lambda_{I_{1}}-\lambda_{T_{1}})}{W_{1}},\quad u_{2}^{*}=\frac{I_{2}(\lambda_{I_{2}}-\lambda_{T_{2}})}{W_{2}}. $$

By using the standard argument for bounds *a*≤*u*_*i*_(*t*)≤*b* for *i*=1,2, we have the optimality conditions (15). □
